# Nanocellulose-Based Inks for 3D Bioprinting: Key Aspects in Research Development and Challenging Perspectives in Applications—A Mini Review

**DOI:** 10.3390/bioengineering7020040

**Published:** 2020-04-29

**Authors:** Xiaoju Wang, Qingbo Wang, Chunlin Xu

**Affiliations:** Laboratory of Natural Materials Technology, Åbo Akademi University, Porthaninkatu 3-5, 20500 Turku, Finland; qiwang@abo.fi (Q.W.); cxu@abo.fi (C.X.)

**Keywords:** nanocellulose, bacterial nanocellulose, cellulose nanofibrils, cellulose nanocrystals, bioink, 3D bioprinting, hydrogel

## Abstract

Nanocelluloses have emerged as a catalogue of renewable nanomaterials for bioink formulation in service of 3D bioprinting, thanks to their structural similarity to extracellular matrices and excellent biocompatibility of supporting crucial cellular activities. From a material scientist’s viewpoint, this mini-review presents the key research aspects of the development of the nanocellulose-based bioinks in 3D (bio)printing. The nanomaterial properties of various types of nanocelluloses, including bacterial nanocellulose, cellulose nanofibers, and cellulose nanocrystals, are reviewed with respect to their origins and preparation methods. Different cross-linking strategies to integrate into multicomponent nanocellulose-based bioinks are discussed in terms of regulating ink fidelity in direct ink writing as well as tuning the mechanical stiffness as a bioactive cue in the printed hydrogel construct. Furthermore, the impact of surface charge and functional groups on nanocellulose surface on the crucial cellular activities (e.g., cell survival, attachment, and proliferation) is discussed with the cell–matrix interactions in focus. Aiming at a sustainable and cost-effective alternative for end-users in biomedical and pharmaceutical fields, challenging aspects such as biodegradability and potential nanotoxicity of nanocelluloses call for more fundamental comprehension of the cell–matrix interactions and further validation in in vivo models.

## 1. Introduction

For developing hydrogel scaffolds that mimic the three-dimensional (3D) architecture of tissue and recapitulate the biological functions, 3D bioprinting stands out to enable the creation of tailor-made tissue engineering scaffolds with individually and digitally designed architecture, and, furthermore, integrating with biological cues to direct cell response in a controlled manner [1,2]. This additive manufacturing technique is based on layered strand-deposition of cell-laden hydrogels and allows digital control over complex geometry (shape, size, and distribution of pores in architecture) to construct functional tissue mimics [3,4]. The technical process of 3D bioprinting engages the extrusion of a feedstock material termed with a bioink, which needs to be in the liquid phase to avoid nozzle clogging, but viscous enough that it holds the printed shape, protects cells during extrusion, and provides the resident cells with an in-vivo-mimicking environment. Minimally, a bioink should exhibit acceptable cell viability while meeting the physical requirements necessary for printing. More desirably, such a multicomponent biomaterial system can provide appropriate surface and adequate space to accommodate cells and other bioactive substances (e.g., cytokine and growth factors) in a biocompatible polymer matrix, as well as direct the crucial cellular activities in three dimensions. Biopolymer hydrogels are the most important representative among a wide array of bioinks. A large variety of natural polymers commonly used in bioink formulation exemplify collagen, gelatin, and hyaluronic acid in the animal-derived resource catalogue, chitosan and alginate in the marine-derived resource catalogue, and polysaccharides derived from various plant resources [3]. As highlighted in the most recent years, nanocelluloses have been established as a renewable constituent in formulating bioinks for hydrogel-extrusion 3D bioprinting [5,6]. Within the scope of biomedical hydrogels for tissue engineering, the exploiting interests on nanocelluloses are mainly aroused by their structural similarity to extracellular matrices (ECM) in terms of both porosity and interconnect framework within the structural hydrogel and fibrous topography of cellulose fibrils somehow analogous to collagen and fibronectin in native ECM, as well as in terms of their excellent biocompatibility of supporting crucial cellular activities, as suggested in a great number of in vitro and in vivo studies [5,6]. Above all, the printability of these biomaterial systems in extrusion-based 3D printing is, in principle, supported by the shear-thinning properties of the nanocellulose hydrogels.

In recent years, our research group has been active in the development of woody nanocellulose-based inks for 3D bioprinting, mainly in the context of biomedical hydrogels for soft tissue engineering applications. Based on the knowledge gathered in this rather specific but cross-disciplinary field, we intend to present the key research aspects within this mini-review, from the preparation of different types of nanocellulose and their respective material properties, to important perspectives in the hydrogel extrusion-based 3D printing processes to consider, and to a discussion on integrating therapeutically relevant functionalities into the nanocellulose matrix from the viewpoint of cell–matrix interactions and delivery of bioactive cues. Lastly, we also attempt to address the challenges that the nanocellulose-based bioinks still face in in vivo applications presently. This review aims for a confined readership among colleagues developing nanocelluloses into various advanced functional materials and researchers from different disciplines of biomedical engineering who are interested in engaging nanocellulose-based inks in their 3D bioprinting approaches. In addition, we acknowledge a few other up-to-date reviews that have also extensively summarised the usage of cellulose and its derivatives in a wider range of scenarios, e.g., the selection of cellulosic materials, the adapted 3D printing techniques, and the underlined applications, to which readers are referred for potential interests [6,7,8].

## 2. Nanocelluloses: Origin, Preparation, and Material Properties on Nano-Scale

In plants, cellulose in the form of para-crystalline microfibrils, as well as nanofibrils, comprises the main load-bearing polymer, which is cross-linked with other macromolecules such as hetero-polysaccharides, lignin, and proteins [9]. The resulting composite confers both strength and flexibility to the plant structure. Keeping in mind the context where a high strength may be offered by the para-crystalline microfibrils and nanofibrils, a large quantity of research work has been laid on the isolation of those fibrils and applying them in various value-added applications [10,11,12]. Different groups of such cellulose nanostructures are thus in focus but with a consensus term of “nanocellulose”, defined as the diameter of the resulted fibril products in one dimension nanoscale. Nanocellulose refers to nanomaterials of three catalogues: bacterial nanocellulose (BNC), cellulose nanofibrils (CNFs), and cellulose nanocrystals (CNCs). CNFs are often noted as nanofibrillated cellulose (NFC) and microfibrillated cellulose (MFC) by researchers in different fields. CNCs referred to nanocrystalline cellulose (NCC) or cellulose nanowhiskers (CNWs) in earlier times. Both CNFs and CNCs can be isolated from biomass resources using top-down approaches that break down the interfibrillated bonds by mechanical disintegration alone or in combination with acidic, enzymatic, or chemical oxidations. However, to note, CNFs contain both crystalline and non-crystalline regions in the fibers, whereas CNCs are generally prepared by hydrolysing the non-crystalline region in acid treatment, thus leaving the crystalline regions. In comparison to CNFs and CNCs, BNCs are prepared in a bottom-up approach via the biosynthesis of cellulose that takes place in a microbial culture through oxidative fermentation. In recent years, cellulosic nanomaterials have shown great potential in biomedical applications owing to their intrinsic characteristics, such as non-cytotoxicity and biocompatibility, high-aspect-ratio material features, strong mechanical properties, and broaden capability for chemical modifications. Furthermore, there is no need to mention the renewable and sustainable nature of nanocelluloses from vast natural resources.

### 2.1. Bacterial Nanocellulose (BNC)

**Figure 1:**
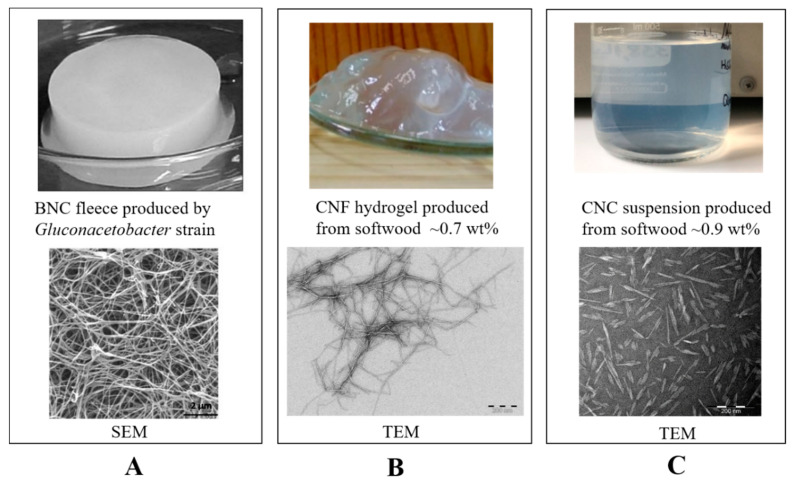
Various nanocellulose products and their microscopic morphology under scanning electron microscopy (SEM) or transmission electron microscopy (TEM) observation: (**A**) BNC (images are reproduced from [10] with the copyright permission from WEILEY-VCH Verlag GmbH & Co.); (**B**) Cellulose nanofibrils (CNFs); (**C**) cellulose nanocrystals (CNCs).

BNC was first discovered in 1886 by A. J. Brown and can be synthesised in the culture medium of glucose and xylose by bacteria such as *Acetobacter xylinum* [13] and *Gluconacetabacter xylinus* [14], yielding similar structures as plant cellulose. In the culture medium, fibrils are synthesised and secreted as exopolysaccharide and thus generate structural hydrogel with interconnected ribbons of around 100 μm in length and 100 nm diameter (as shown in Figure 1A) [10]. This catalogue of microbiologically derived nanomaterials possesses outstanding features of high purity (free of pectin, hemicellulose, and lignin), degree of polymerisation (up to 10,000), and crystallinity (>85%) of the cellulose microfibrils in BNCs [15]. Importantly, the large hydrophilic surface area originated from cellulose microfibrils endows the BNC hydrogel with excellent water retention capability. Meanwhile, BNCs in the wet state show strong mechanical properties and good flexibility [16]. They are recognised to be highly compatible biomaterials for constructing tissue engineering scaffolds [17] and have been successfully applied for long in a number of biomedical applications such as wound dressing [18], artificial skin [19], vascular and cartilage implants [20,21]. Xylos Corporation (USA) has developed a BNC-based product, XCell, for choric wound dressing, which has been commercialised since 2003 [22]. A series of CELMAT^®^ products that based on BNC are available as facial and eye masks and wound dressing from BOWIL Biotech Ltd. (Poland). However, process factors such as the high cost of glucose as the carbon source and the labor-intensive and low-productivity culture process to yield BNC still restrict the up-scaled production of BNC hydrogels for cost-effective commercialisation [23].

### 2.2. Cellulose Nanofibrils (CNFs)

Cellulose nanofibrils (CNFs) can be produced from biomasses such as wood pulp. CNFs display as fibrils with diameters of 5−60 nm and lengths of approximately up to a micrometer (as shown in Figure 1B). Notably, the preparation procedures largely impact on the surface chemistry of the resultant CNFs. At the same time, these surface-modified groups offer an untapped possibility for further functionalisation of CNFs. TEMPO-mediated oxidation, in combination with mechanical defibrillation, produces well-fibrillated CNF with defined surface chemistry of abundant carboxylic groups and a small aldehyde content [24]. Periodate oxidation results in “dialdehyde” CNF by opening the glucose ring on the C2 and C3 sites in the cellulose molecular chain [25]. These flexible CNFs with a high aspect ratio give a gel-like consistency in aqueous suspensions at above a certain dry matter content, depending on the chemical nature and charge density of surface-modified groups on the nanofibrils (as shown in Figure 1B). In the past decade, CNFs have been intensively investigated in versatile applications of cosmetics, pharmaceutics, and biomedical devices [5,26,27]. At present, a CNF product of medical-grade is commercially available from UPM Biomedical (Finland) under the trademark of GrowDex^®^ as a generic 3D cell culture matrix, which is produced with mechanical defibrillation from a sustainable wood resource.

### 2.3. Cellulose Nanocrystals (CNCs)

Cellulose nanocrystals (CNCs) are prepared by digestion of the cellulosic materials in strong acids (e.g., sulfuric acid or other mild mineral acids) to hydrolyse the amorphous region in cellulose microfibrils, followed by mechanical defibrillation. Hence, wood-derived CNCs that are dispersed in aqueous media display as nanorods of a high aspect ratio (9–50), with a diameter of 3–10 nm and length of 50–500 nm (Figure 1C). More interestingly, CNCs form a chiral nematic liquid crystal phase under the circumstance of controlling the ionic strength of suspension or applying a strong magnetic field [28]. Such features as extremely high stiffness of single nanorod (high crystallinity) and ordered alignment of CNCs in the liquid crystal phase make them attractive nanofillers in preparation of reinforced composite matrices with mechanically anisotropic features [29]. Meanwhile, the surface of CNCs is activated with carboxylates or sulfates, which result from and are dependent on the acidic process adopted. Similar to CNFs, CNCs can also be prepared by comparatively sever periodate oxidation that results in “dialdehyde” on the C2 and C3 sites in glucose units [25]. The surface-modified carboxylates and aldehyde groups facilitate various chemical modification routes to carry out the derivatisation of CNCs. CNCs show great potential for a wide range of applications such as composite reinforcements, flocculants, and certain biomedical areas [30,31,32]. For instance, nanosized CNCs may pass the cell membrane and thus have found applications in drug delivery and targeted imaging as nanomedicines [33]. 

These above-described nanocellulose products (BNCs, CNFs, and CNCs) are displayed in Figure 1A–C, along with their representative scanning electron microscopy (SEM) or transmission electron microscopy (TEM) images for microscopic observation.

## 3. Nanocellulose-Based Bioink: Rheological Properties and Cross-Linking Strategy vs. Ink Fidelity

**Figure 2:**
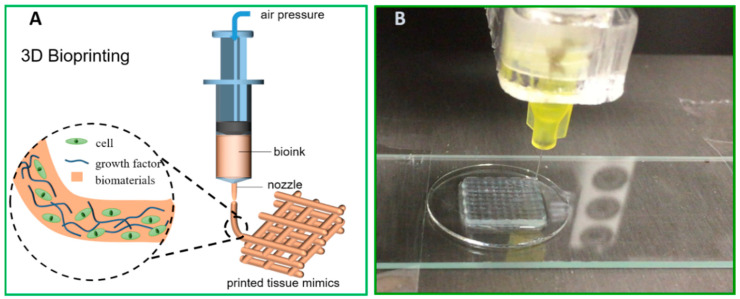
(**A**) Schematic illustration of direct ink writing (DIW) 3D bioprinting and (**B**) DIW printing of TEMPO-oxidised CNF ink at a dry matter content of 1 wt%.

Direct ink writing (DIW) is the most common extrusion-based additive manufacturing technique used in 3D bioprinting. As illustrated in Figure 2A, this technique employs the cell-laden hydrogel as a feedstock bioink. In printing, the air pressure or the displacement of syringe piston results in stress inside the nozzle on the printer head, where the viscosity of ink decreases and flows through the dispensing nozzle. Once the ink is deposited and the stress disappears, the laid-down hydrogel relaxes and forms a filament of solid gel. Step-by-step, a digitally defined 3D object can be built-up by the layered strand-deposition of the ink.

Speaking of appropriate material properties demanded for a bioink, the rheological properties of these biomaterial systems are highly relevant. At first, a shear-thinning behaviour is a must to validate the extrusion-based printing, which enables the ink to pass through the narrow nozzle with low resistance under a certain shear. The yield stress and the shear-thinning response of a bioink are always studied to predict a window of printer operating parameters. Secondly, the viscoelastic property of ink is sufficient to provide ink fidelity when the hydrogel network rapidly recovers elasticity after relaxation, which is technically critical to prevent ink viscous flow and collapse of the wet printed object. In principle, the rheological behaviours of various nanocellulose types largely differ depending on the morphological (size and shape of stiff nanorods in CNCs vs. interconnected nanofibrils in BNC and CNF) and the surface-chemical (charge and other functional groups) attributes of the nanocellulose product. A comprehensive review by M.A. Hubbe and his colleagues is referred to for a detailed understanding of the rheology of nanocellulose-rich aqueous suspensions [34]. For the CNC that is surface-modified with sulfate half-ester groups, its water-like suspensions at low concentrations have a low viscosity (not printable). When such CNCs are added as reinforcing nanofiller in binary systems with other water-soluble polymers, they function as rheological modifiers. For instance, in a binary CNC/alginate bioink system, the addition of CNCs into alginate solution resulted a viscoelastic system with a higher storage modulus (G’) than the loss modulus (G’’), which consequently assured printability as well as enhancing ink fidelity, compared with the liquid-like alginate solution [35]. The pristine CNC suspensions were shown to be printable only at concentrations above at ~10 wt%, when the shear-thinning behaviour and viscoelastic property of the system meet the rheological demand for a specific DIW setup (e.g., pressure inside syringe and diameter of the extruding nozzle) [36]. These properties are attributed to the interactions between adjacent particles at high concentrations. When disintegrated and suspended in aqueous solution, the BNC fibrils may behave like “flocs” in suspension and can also be oriented under certain shearing conditions to resemble the liquid crystals [37,38]. The dispersions of BNC fibrils show a shear-thinning behaviour and a higher viscosity compared to CNC suspension at the same concentration. In 3D bioprinting, BNC fibrils or oxidised BNC fibrils were printed in blends with alginate for its reinforcing effect [39,40]. The printing of BNC fibrils alone was less practiced, probably due to the lack of cross-linking sites on BNC fibrils to support good performance in terms of ink fidelity. For CNFs, the pretreatments in CNF preparation regulate both the morphology and surface functionality of the CNF products, which in turn determine the interactive forces between nanofibers in the suspensions. The viscosity of carboxymethylated CNF- and TEMPO-oxidised CNF-based inks increases rapidly with increasing the concentration. Both types of CNFs present as a stable hydrogel with strong viscoelastic modulus even at a low concentration of around 1 wt%. This is mainly a result of the electrostatic repulsion among negatively charged carboxylate (–COO^−^) groups on CNF fibers. For instance, the TEMPO-oxidised CNFs give a hydrogel-like viscosity when the dry matter content is above a critical concentration in range of 0.5~1 wt%, depending on the charge density on nanofiber resulted from the pretreatment of TEMPO-mediated oxidation. Experimentally, in a dry matter content range of 1~5 wt% in the ink, the shear-thinning rheology and viscoelastic property of these CNFs allow a continuous extrusion of hydrogel filament [41]. 

More importantly, in situ cross-linking is further relied upon to strengthen the hydrogel network and to generate the adhesion between adjacent layers in order to guarantee the integrity of complex geometry in fabrication. To cross-link the cellulose fiber surface, the strategies can be either physically cross-linked through transient weak interactions (e.g., Van der Waals force, ionic interaction, hydrogen bonding, and hydrophobic interaction) or chemically cross-linked through permanent covalent bonds. Common reaction mechanisms to induce chemical cross-linking in hydrogel systems exemplify condensation, such as the Schiff’s base formation and free-radical polymerisation catalysed by enzymes or photo lights [42,43,44]. These above-mentioned cross-linking strategies have been implemented in the nanocellulose-based inks, aiming to facilitate continuous printability and improve shape fidelity. Physical cross-linking offers a facile and easily applicable approach. Ca^2+^ can cross-link the CNF network by complexing the –COO^−^ groups on the fiber surface. For instance, a 5-wt% CaCl_2_ solution was used in droplets during printing the TEMPO-oxidised CNF ink (1 wt%) to gain good shape fidelity via the ionic cross-linking, as shown in Figure 2B [41]. In DIW printing engaging a binary CNF/alginate ink, Ca^2+^ is extensively used as a cross-linker to give good ink performance in terms of the rheological properties, compressive stiffness, and shape fidelity of the printed inks, mainly owing to the presence of large content of –COO^−^ groups in the alginate molecular [45]. Meanwhile, ionic cross-linking is weak and transient, which is sensitive to a micro-environmental alternation of pH or ionic strength, whereas, chemical cross-linking creates comparatively robust and strong hydrogels with covalent bonds. In this scenario, enzymatic cross-linking and UV-induced cross-linking are engaged when the CNF is in cooperation with an auxiliary biopolymer to formulate compatibly blended binary ink systems. For instance, the intrinsic affinity of cell wall hetero-polysaccharides for cellulose has inspired their use to modify the surface chemistry and mechanical properties of cellulosic materials. As a pioneer for the biomimetic CNF/wood-derived hemicellulose bioinks, K. Markstedt and P. Gatenholm et al. established an enzymatic approach of utilising horseradish peroxidase (HPR) to cross-link the binary hydrogels of CNF/tyramine-modified xylan [46] or CNF/tyramine-modified galactoglucomannan (GGM) [47]. There, HPR catalysed the bond formation between phenolic groups in tyramine-modified xylan or tyramine-modified GGM to result in the cross-linking of the hydrogel network in the printed construct. Photo cross-linking is another easy-to-apply approach as it avoids wet chemistry as well as providing quick gelation, and more importantly, acceptable biocompatibility to the seeded cells. This strategy was applied in binary hydrogels of CNF with a methacrylated biopolymer as the auxiliary polymer, such as in our studies on low-concentration DIW inks of TEMPO-oxidised CNF with methacrylated gelatin (GelMA) [48] and TEMPO-oxidised CNF with methacrylated GGM [49]. In these cases, a UV-LED that is built-in with a DIW printer and moves along with the printing head initialises the radical polymerisation of methacrylates, while the wet filament is laid down to result in an excellent ink fidelity even in the cases of low-concentration inks.

For 3D bioprinting, assessment of ink fidelity is a critical aspect in terms of supporting the layer stacking without the deformation or collapse of overhanging filaments as well as in terms of avoiding compromising of the printing resolution caused by possible fusion between adjacent wet filaments [3,50]. In other words, it reflects how much the printed construct would be able to replicate the digital design. Experimentally, ink fidelity can be determined with regard to the ratio of line width to nozzle diameter (line resolution), the number of layers until collapse, or the curvature of printed lines for constructing complex geometry. For CNFs, the dry matter content in the hydrogel is typically limited up to around 5 wt%, dependent on the fiber dimensions and surface chemistry of the nanofibers. In general, a higher dry matter content used in the CNF ink aids better ink fidelity. Then, higher shear stress has to be applied to yield the ink flow, which might be less desirable from the cell viability viewpoint. When a low-concentration CNF-based ink is printed, an in situ cross-linking strategy has to be integrated to provide strong hydrogel filament in support of a good ink fidelity, as earlier discussed. Meanwhile, the swelling behaviour originating from the hydrophilic nature of cellulose has an impact on the printing resolution. J. Leppiniemi et al. studied the influence of cross-linking on swelling behaviour of 3D printed CNF/alginate grid by soaking the cross-linked and uncross-linked grid into phosphate-buffered saline (PBS buffer). The shape of the cross-linked grid still remained after 24 h (no significant changes on the outer edge), whereas the uncross-linked grid lost its structure in one hour and turned into gel after 24 h [51]. Also, the shape resolution of the cross-linked CNF/alginate grid was sensitive to the variation of ion strength in the soaked media since its swelling behaviour was regulated by ionic cross-linking between Ca^2+^ and the –COO^−^ groups in alginate [51]. W. Xu et al. studied the influence of cross-linking density on the resolution of the printed GelMA/CNF grid in PBS buffer. The result showed the UV cross-linking could lead to a higher printing resolution and the resolution was enhanced as the increase of cross-linking density [48].

When optimising the ink fidelity, apart from regulating the material properties of the ink itself, as discussed above, other factors should also be taken into consideration, including printing parameters (printing speed, nozzle height, flow rate, and printing path) and the impact of the addition of cells. Actions in optimising printing fidelity of nanocellulose-based inks with respect to printing parameters are still scarce, as challenged by the highly time- and material-consuming lab practice. J. Göhl and his colleagues have adapted the computational fluid dynamics (CFD) tool to stimulate the ink fidelity feature by comparing a 3-wt% binary ink of CNF (non-charged) blended with alginate and a 4-wt% carboxymethylated CNF ink [50]. The CFD simulation helped to understand how various printing parameters affected the line resolution as well as how the viscoelastic stress was distributed throughout the printed filament, which is important for cell viability in 3D bioprinting.

## 4. Cell–Matrix Interactions and Delivery of Bioactive Cues in Hydrogel Scaffold Fabricated by 3D Bioprinting of Nanocellulose-Based Bioinks

Biocompatibility of cellulose nanomaterials is the primary aspect to investigate as a biomaterial in use. BNC offers excellent biocompatibility. It is a catalogue of well-accepted biomaterials as topical wound healing dressing in treating burns and severe wounds [18,22], as it can offer excellent water retention capability and favorable bioactivities, such as lowering inflammatory response and promoting the fibroblast proliferation [20]. For CNFs and CNCs produced from various resources and preparation methods of different kinds, the non-cytotoxic features of them were verified with a few cell lines in a large number of in vitro cell culture studies. Good biocompatibility of woody nanocellulose was verified with respect to crucial cellular activities of fibroblast growth and proliferation [52]. A study by Y. Lou et al. showed that CNF hydrogels three-dimensionally supported the crucial cellular activities in the culture of human pluripotent stem cells [53]. It was also found in our own studies that the chemical and structural features of CNFs had an impact on the mechanical properties of thus-prepared matrices and further regulated the cell viability in the culture of fibroblasts [54,55]. Furthermore, the binary system of CNF and alginate has been validated to be non-toxic in the culture of various cell lines, e.g., human nasoseptal chondrocytes [45], human-derived induced pluripotent stem cells [56], and fibroblast cells [45]. Collagen and gelatin, the most common biocompatible polymers, are often blended with nanocellulose to create a more biocompatible 3D culture platform, as these biopolymers contain the RGD-moieties that promote cell attachment to the hydrogel matrix [48,57].

Moreover, a bioink system is desired to meet a number of criteria in order for the constructed tissue engineering hydrogel scaffolds to function properly both in vitro and possibly in vivo. In vivo, the ECM provides a microenvironment with proper composition, structure, and stiffness, which is critical for biological processes such as cell adhesion, migration, differentiation, proliferation, and survival [58]. From this perspective, the ECM-mimicking 3D culture platforms would allow us to investigate cell and tissue physiology and pathophysiology in in vitro cell culture to be most relevant and reliable when compared with in vivo conditions [59]. To truly mimic the ECM, the man-made matrices are desired to have innate structural similarities with physiological matrices in the body tissues and to support the most crucial cellular activities, such as cell attachment, proliferation, and subsequent tissue formation [60,61]. First of all, the mechanical characteristics such as ECM rigidity and alignment or organisation play essential roles in various biological processes [62]. For example, A.J. Engler et al. identified that the matrix elasticity for stem cell culture was able to direct the differentiation of human mesenchymal stem cells (hMSCs) toward specific fates [63]. Regarding this perspective, the stiffness control in the ink matrix is a very important biofunctionality to be endowed with. Secondly, cells interact with the biochemical and biophysical cues within their surrounding microenvironment, and such interactions collectively regulate cell behaviour, function, and fate in vivo [58]. Therefore, it is highly demanded to create biofunctionalites of the man-made matrices to spatiotemporally deliver a variety of bioactive cues, aiming to promote the bidirectional crosstalk between the scaffold microenvironment and the resident cells in tissue engineering scaffolds.

### 4.1. Versatile Cellulose Chemistry to Improve Matrix Reactivty

Various chemical modifications of cellulose are often needed to improve the accessibility of cellulose fibers for adding further functionality to the nanocellulose matrix. Cellulose possesses enormous hydroxyl groups that are amendable for further functionalisation. The chemical modifications are heterogeneous when the nanocellulose presents as BNC, CNF, or CNC. Through reacting with the hydroxyl groups, both cellulose ethers and esters can be prepared by different reaction routes. The functional groups/the tethered moieties are established on the nanocellulose surface, which supports the applicability of nanocellulose hydrogels as ECM-mimicking matrices. In a state-of-the-art study by G. Siqueira et al., the acetylation of CNC with methacrylic anhydride via hydroxyl groups was carried out to result in (hydroxyethyl)methacrylated CNCs, which were established as the foundation of a CNC-reinforced and polyurethane acrylate oligomer-based ink for DIW printing of textured cellular architectures aided by photo cross-linking [36]. 

Moreover, the active sites such as carboxylates, aldehydes, and sulphates that were introduced by the pretreatments could also be used directly for further functionalisation. Studies on negatively charged, TEMPO-oxidised nanocellulose have been much in focus due to its gelling property, which can be further enhanced by additional multivalent metal ions such as Ca^2+^. A double cross-linking approach, where TEMPO nanocellulose was cross-linked during printing by addition of an aqueous Ca^2+^ solution, was followed by a post-printing chemical cross-linking with 1, 4-butanediol diglycidyl ether [41]. These printed scaffolds were proven to be stable in PBS buffer for over three months. Carboxylmethylation is another well-established approach to introduce carboxylates to cellulose, which is an effective pretreatment method in producing negatively charged CNFs [64]. The “dialdehyde” resulted in the CNF and CNC from pretreatment of periodate oxidation is actively ready for further chemical derivatisations, e.g., reductive amination. For instance, the dialdehyde-modified CNF reacted with collagen to result in a biocompatible composite platform [65]. In another study, the “dialdehyde”-modified CNCs reinforced the polysaccharide hydrogel of carboxymethyl cellulose–hydrazide (CMC–NHNH_2_) and dextran–aldehyde (DEX–CHO), being chemically cross-linked within the structural hydrogel network via reductive amination [66]. 

### 4.2. Cell–Matrix Interactions

In order to support the cell-matrix interactions better and possibly to add the relevant biofunctionality for a desired therapeutic effect, e.g., osteogenic or angiogenetic effect, biomaterials can be engineered on various aspects, such as surface morphology and chemistry of the biomaterials, micro-engineered mechanical stiffness in the matrix, and stimuli-responsive property of the matrix material. When it comes to the chemical modification to the nanocellulose, it is critical that the reaction medium and reagents selectively used in the chemical modification, as well as the added functionalisation, still guarantees the biocompatibility of biomaterials.

Prof. Paul Gatenholm and his colleagues from Chalmers University of Technology (Sweden) have extensively investigated the interaction of *Gluconacetobacter xylinus*-sourced BNC solely or in binary hydrogel scaffolds with different cell lines such as SH-SY5Y neuroblastoma cells [67], human-derived induced pluripotent stem cells [56,68], and chondrocytes [20,69] towards tissue engineering applications. The mechanical strength of scaffolds was tuned by varying the BNC content to meet the requirement by the targeted tissue [70]. The surface chemistry of the BNC scaffold was also tailored by a coating of collagen to improve cell adhesion, growth, and differentiation [67]. The cell-laden 3D-bioprinting of BNC-based scaffolds has brought up promising solutions for tissue repair [71,72]. A recent review by A. Sionkowska et al. presented recent advances of the most studied medical applications of BNC [73]. 

For the CNF hydrogels, it is well established that the surface chemistry resulted from various preparation methods has a large impact on cell–matrix interactions. Hence, the CNF hydrogel matrices undergone chemical post-modifications particularly need to be carefully evaluated in cell culture studies prior to formulating bioink. As wound healing and soft tissue engineering are the most discussed end applications for CNF-based bioinks, the fibroblast is the most used cell line in in vitro culture for evaluating the cytotoxicity of CNF hydrogels [52,54]. In addition, cancer cell lines and stem cells were also tested in a number of in vitro studies on evaluating the CNF hydrogels as cell culture platforms [52,53,54]. L. Alexandrescu et al. created different surface chemistry on cellulose fibers by either enzymatic pretreatment or TEMPO-oxidation in combination with mechanical defibrillation, resulting in CNF free of charge or with anionic charge, respectively [52]. Both types of CNFs showed excellent compatibility and strong cell–matrix interactions in supporting the attachment, proliferation, and growth of 3T3 fibroblasts. The same study also investigated two post-treatment approaches to tune the surface properties, polyethyleneimine (PEI)-cross-linking and sorption of cetyltrimethylammonium bromide (CTAB), both of which induced reduction in cell viability. Thus, when tailoring properties of scaffolds, one needs to keep in mind that cells are sensitive to surface alternation that they will be in contact with. 

For the CNF hydrogel produced with the pretreatment of TEMPO-mediated oxidation followed by high-pressure homogenisation, it has demonstrated excellent DIW printability in quite a few studies. The narrow size distribution in terms of nanofiber length in a homogeneous hydrogel phase guarantees a consistent ink flow without clogging the nozzle while being extruded [41]. Speaking of the CNF hydrogel preparation, the charge density of –COO^−^ introduced by TEMPO-oxidation to the nanofiber plays a decisive role in fiber disintegration to obtain the nano-dimensional fibrils [24]. Meanwhile, the charge density of –COO^−^ largely impacts the biocompatibility of TEMPO-oxidised CNF with respect to the growth of fibroblasts and Hela cancer cells inside the hydrogel. An intermediate surface charge level of around 1 mmol/g was suggested to provide good biocompatibility in favor of the cell–matrix response [52,54]. Furthermore, the negatively charged COO^−^ groups on CNF is an important factor to consider in the case of formulating a composite ink with a second biopolymer. When formulating the composite ink of TEMPO-oxidised CNF/GelMA, GelMA had to be kept less than 1 wt% in the binary system containing 1 wt% TEMPO-oxidised CNF in order to avoid the phase separation caused by the ionic interaction between TEMPO-oxidised CNF and GelMA, and the ink homogeneity was thus retained [48]. 

**Figure 3:**
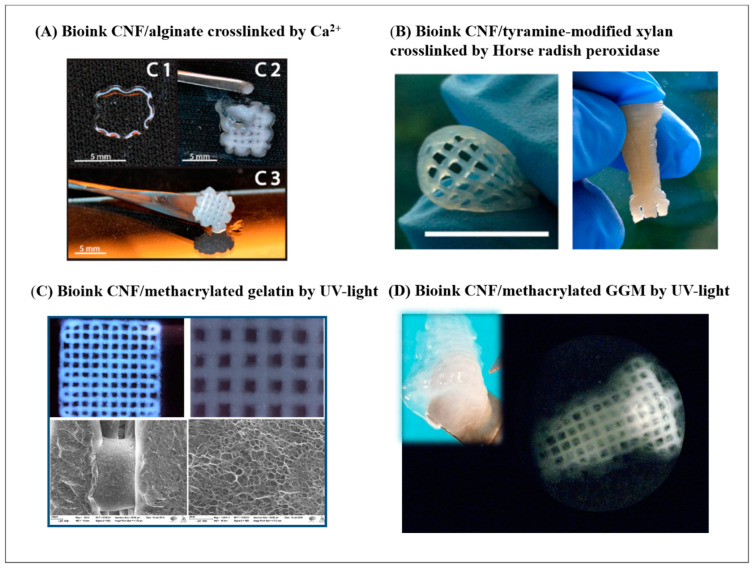
State-of-the-art CNF-based bioinks engaging different cross-linking strategies: (**A**) bioink CNF/alginate cross-linked by Ca^2+^, as presented in [45] (copyright permission from American Chemistry Society); (**B**) bioink CNF/tyramine-modified xylan cross-linked by horseradish peroxidase, as presented in [46] (copyright permission from American Chemistry Society); (**C**) bioink CNF/methacrylated gelatin by UV light, as presented in [48] (under CC-BY licence); (**D**) bioink CNF/methacrylated gelatin by UV light, as presented in [49] (under CC-BY licence).

To meet the requirements of desired matrix stiffness and shape fidelity, a cross-linker is often introduced in ink formulation. Various strategies as dispicted in Figure 3A–D, such as alginate in combination with Ca^2+^ and polymeric methacrylates as UV-curable cross-linkers, could be applied in the nanocellulose-based formulation to provide the control means over the mechanical properties of the bioinks. Then, the cell–matrix response is evaluated in various cell cultures by seeding the cells into 3D-printed porous hydrogel scaffolds or cell-laden bioprinting. Different cell lines were used to investigate the response of cells in those scaffolds with tuned mechanical property: fibroblast, breast cancer cells, human neuroblastoma, different stem cells (seen as in Table 1). In our earlier approach inspired by the intrinsically high affinity between cellulose and hemicellulose in plant cell walls, hemicelluloses (xylan, GGM, and xyloglucan) were engaged as physical cross-linkers to prepare hemicellulose-reinforced TEMPO-oxidised CNF hydrogels [74]. There, it was demonstrated that the incorporation of xyloglucan significantly increased the modulus and yield stress of the aerogels and correspondingly supported the cell functions seeded in the reinforced CNF hydrogel matrix in comparison with the one-component CNF hydrogel [74]. As lately investigated in the double cross-linking approach applied in DIW printing, the compressive Young’s modulus of a one-component TEMPO-oxidised CNF hydrogel scaffold resulted in a range of 3 to 8 kPa that suits the attachment of fibroblast cells. Further cell tests confirmed that the hydrogel rigidity had a clear impact on the cell–matrix response: the proliferation of fibroblast was promoted with increased hydrogel stiffness within the studied range [41]. In this study, the stiffness variation was resulted only by chemically cross-linking the hydroxyl groups in a one-component CNF hydrogel, which affects the least the surface chemistry of matrix, and this makes the correlation between the mechanical properties of the hydrogel matrix and cell–matrix response easy to demonstrate. In the binary ink formulations where the methacrylated natural polymers are UV cross-linkers for mechanical stiffness control over the CNF hydrogel, the introduction of a secondary component also alters the chemical nature and surface physiochemical features (surface roughness and porosity) of the hydrogel matrix in different means [48,49]. Together with the mechanical stiffness of the hydrogel matrix, these factors together regulate the cell–matrix response. In the fields of 3D bioprinting, the methacrylated biopolymers (e.g., GelMA) have become popular in formulating various photo cross-linkable bioinks. It is still worth noting that the cytotoxicity of such systems on the resident cells may potentially originate from the free radicals that are generated by the photo-initiator when activating the cross-linking of methacrylate groups. Herein, the selection of biocompatible photoinitiator and degree of substitution (DS) of methacrylate in biopolymer) are important aspects to consider. M.J. Majcher et al. showed that methacrylated starch nanoparticle-based hydrogel showed lower cytotoxicity with a DS lower than 0.10 [75]. Owing to its hydrophilic property, 2-hydroxy-4′-(2-hydroxyethoxy)-2-methylpropiophenone (Irgacure 2959) is widely accepted as a biocompatible photoinitiator for such photo cross-linkable bioink formulations in the concentration range of 0.03%–0.1% w/v [76].

CNCs obtained by acid hydrolysis are characteristics of negatively charged surface groups (–OSO_3_^−^ or –COO^−^, depending on the treatment acid that is adopted) and nanorod-like morphology. In the context of 3D bioprinting, they are more often seen as reinforcing nanofillers in formulating composite hydrogel bioinks used in bone/cartilage regeneration, owing to their nanorod-like morphology and extraordinarily high stiffness [77]. Importantly, S. Dong et al. performed 3-(4,5-dimethylthiazol-2-yl)-2,5-diphenyltetrazolium bromide (MTT) and lactate dehydrogenase (LDH) assays to study the cytotoxicity of CNCs against nine different cell lines [78], showing no cytotoxic against any of these specific cell lines over the tested concentration range. Furthermore, the unique property of ordered alignment of CNCs in the liquid crystal phase makes them interesting as reinforcing nanomaterials to result in the anisotropically mechanical properties of the matrix [30]. A matrix possessing ordered structure is particularly of interest for tailoring gradients in alignment with or against the order. As reported by K.J. De France et al. [29], the magnetic field-induced alignment of CNCs was successfully translated into a nanocomposite hydrogel based on hydrazone cross-linked poly(oligoethylene glycol methacrylate) (POEGMA) and physically incorporated CNCs after injection, which consequently endowed the hydrogel matrix with anisotropic properties. Meanwhile, the anisotropically mechanical property is directive for the motility and migration of cells, such as skeletal muscle myoblasts in the repair of muscle tissue. The nanocomposite hydrogel of POEGMA/CNCs promoted the differentiation of resident skeletal muscle myoblasts into highly oriented myotubes in situ [29,79]. According to J.M. Dugan et al., oriented surfaces of highly charged CNCs prepared using a spin-coating method also induced contact guidance in skeletal muscle myoblasts [79]. A bit surprisingly, fibroblasts tended not to adhere to the CNC coating in comparison to different types of CNF (no-charge CNF and negatively charged TEMPO-CNF) coatings, as recently reported in our study [80]. This might indicate the effect of material stiffness and nanoscale morphology of nanocelluloses on the cell–matrix interactions for different cell types. 

### 4.3. Delivery of Bioactive Cues in the Nanocellulose-Based 3D Bioprinting

As above-mentioned, it is highly demanded to create molecular functionalities of the hydrogel matrix to spatiotemporally deliver a variety of bioactive cues within the tissue engineering scaffolds. More specifically, the hydrogel scaffolds need to be able to act like natural ECM being a carrier for bioactive substances, such as growth factor or bioactive drugs that can regulate the cell behaviour as desired. Owing to the high surface area-to-volume ratio of nanocelluloses, adsorption and entrapment of active substances into their porous structure as such or after surface modifications could enable high levels of drug loading and binding. S. Chatterjee and C. P. Hui recently reviewed the stimuli-responsive polymers, mainly including chitosan, cellulose, and gelatin, imparting sensitivity to act in responding to temperatures and pH conditions for their respective applications in drug delivery [81]. Those hydrogel systems are usually composed of natural polymers and responsive polymers, e.g., poly(N-isopropylacrylamide) (pNIPAAm) by blending or covalent bonding. Another review has summarised the state-of-the-art in chemical modifications of cellulose, lignin, and other wood components via atom transfer radical polymerisation, which broadens their potential applications in medicine and pharmacy as stimuli-responsive micelle delivery systems and gene carriers [82].

The modification of cellulose and other biopolymers often allows better delivery of growth factors and other active drugs with increased binding ability. Thus, those bioactive cues can be directly formulated into the inks. For example, growth factor was blended in alginate/carboxymethyl cellulose formulations for the 3D printed thin films, and significantly improved cell viability was detected for films with incorporated growth factors in the cell tests with the most abundant skin cell types (keratinocytes and fibroblasts) [83]. 

M. Ojansivu et al. have developed an interesting composite ink containing a polymeric matrix of CNF/alginate/gelatin and bioactive glass (BaG) microparticulates for the fabrication of in vitro tissue equivalents that are proposed for studies of bone/or cartilage regeneration [84]. In their system, CNF regulated the rheological properties of the composite ink to facilitate DIW printing. BaG microparticulates were integrated as a therapeutic functionality carrier as the BaGs have high osteogenic bioactivity attributed to the released therapeutic inorganic ions (Si, P, and Ca) functioning as bioactive cues in vivo [85]. In the printed hMSCs-laden constructs, the BaG microparticulates embedded in the ink were confirmed to stimulate the early osteogenic commitment of the resident hMSCs [84]. 

With an attempt to summarise the ink formulations that have used nanocelluloses as the major component other than an auxiliary one, the authors present the most recent studies in the literature in Table 1. These studies are discussed with respect to ink composition, printing approach, indications on cell behaviours in cell culture, and biomedical applications, as highlighted. 

## 5. Challenges and Perspectives for Nanocellulose-Based Inks

In the research fields embracing natural polymers as biomaterials, nanocelluloses of various types have gained numerous interests as nanoscaled components for formulating sustainable bioinks. Their potential applications are seen not only in constructing 3D cell culture platform for drug screening and cancer research but also in fabricating skin tissue mimics and composite hydrogel scaffolds used in cartilage/bone tissue reparation via either scaffold-printing or cell-laden 3D bioprinting. On the one hand, as biomaterials are mainly evaluated in in vitro cell culture at the present stage, excellent biocompatibility and strong cell–matrix interactions of nanocelluloses have been highly praised in a number of research studies. As above-mentioned, these outstanding properties of nanocellulose-based bioinks are attributed to their structural similarity in fibrous morphology with the ECM, as well as their nano-sized material features that offer large surface areas and a highly compatible chemistry nature for the accommodated cells to interact with. On the other hand, the popular acceptance of nanocellulose-based bioinks among the potential end-user society, mainly referring to cell biologists and medical surgeons, has been challenged by their concerns on the in vivo biodegradability and validation of in vivo nano-safety for cellulose nanomaterials to date.

Since human beings do not have specific enzymes (e.g., cellulases) that can break down the nanocellulose in vivo, it is not well accepted to engage the nanocellulose itself or the composite material with nanocellulose as the main constituent in implant manufacture where the biomaterials are highly desired to be bioresorbable after tissue healing or organ repair. Some studies showed that oxidised cellulose has the potential to be degraded by the human body, owing to their weak resistance to hydrolysis [26]. A. Rashadet et al. studied the degradation profile of TEMPO-oxidised and carboxymethylated CNF scaffolds in vitro. The result showed 6.7% and 6.5% weight loss of the scaffold after 90 days, respectively [86]. In contrast, periodate-oxidised BNC showed faster degradation kinetics. W. Czaja et al. treated the pressed BNC sheet with pre-γ-irradiation followed by sodium periodate oxidation and the oxidised BNC sheet showed an in vitro degradation rate of 85% in 7 days. The further in vivo test showed the degradation occurring in the first 2 to 4 weeks [87]. 

A limited number of nanotoxicology studies have addressed the toxicological effect of CNFs and CNCs in in vivo animal models, such as zebrafish [88] and rat [89], although no significant risks were indicated for acute toxicity in small quantities [90]. Meanwhile, long-term nanotoxicity of nanocellulose is another concern that is closely associated with the in vivo degradability problem and it is an important research aspect that still awaits a large number of assessments in in vivo models. Above all, the most intriguing question present for material scientists in basic research is how to chemically modify or engineer the nanocellulose materials to make it self-degrade naturally in the human body.

To date, the most promising applications for CNF-based bioinks can be seen in the fabrication of skin tissue mimics as culture platforms for in vitro studies focusing on cell–cell interactions in elucidating the molecular mechanism of disease or cellular response to the bioactive substances in drug-screening. As one of the frontier players in the commercialisation of medical-grade nanocellulose products (from wood resources), UPM Biomedicals has newly launched a medical-grade CNF hydrogel with the trademark of GrowInk^TM^ as a non-animal-derived bioink, as well as a CNF-based wound dressing product of FibDex^®^ for the European market. As supported in a clinical trial of small group of patients, FibDex^®^ is claimed to provide a favorable environment for the healing of wound to occur [91]. Swedish bioprinter supplier CELLINK is also commercialising CNF/alginate bioink as accessory kits for the use in their bioprinter series. Their CNF/alginate bioink was used to fabricate human cartilage construct with chondrocytes and stem cells co-cultured inside the hydrogel. As evaluated in an in vivo mice model, the matrix supported not only the proliferation of chondrocytes but also the secretion of glycosaminoglycans and collagen II by the chondrocytes [92]. In the near future, with more comprehension of cell–matrix interactions from fundamental studies and validation of nanocellulose products in in vivo models, the nanocellulose-based bioink is anticipated to offer a more sustainable and cost-effective alternative for end-users in biomedical and pharmaceutical fields.

**Table 1:** Overview of recently developed nanocellulose-incorporated ink formulations and their in-vitro cell culture studies.

Nanocellulose Type	Composition of Inks	Printing Approaches	Cell Lines	Cell Study Results	Potential Applications	References
Bacterial CNF	CNF + silk + gelatin + glycerol	Hydrogel DIW	L929 fibroblasts cells	The in vitro evaluation showed that the composite scaffolds had excellent biocompatibility, while the in vivo results demonstrated that the hierarchical pore structure was beneficial to the ingrowth of tissue	Repair of soft tissues	[93]
CNF	CNF + cross-linkers (CaCl_2_, Chitosan oligosaccharides, Poly-l-lysine, protamine)	Inkjet spray, cell-laden	Mouse fibroblasts (NIH3T3), human embryonic kidney cells (293A), and human newborn foreskin fibroblasts (Hs68)	cell viability, metabolic activity, and collagen type I secretion were evaluated in the printed objects	Skin tissue mimics	[94]
CNF	CNF + CMC/Alginate	Hydrogel DIW	Human primary pancreatic cells	Promoted cell adhering, aggregation, migration, and support long-term growth of pancreatic cell	Cell culture and disease study	[95]
CNF	CNF + alginate	Hydrogel DIW, cell-laden	Mouse mesenchymal stem cell line C3H10T1/2	The cells accumulate more lipids and have increased gene expression of adipogenic marker genes PPARγ and FABP4 than cells cultured using standard 2D method	3D cell culture of adipocytes	[96]
Enzymatic CNF	CNF + alginate	Hydrogel DIW, cell-laden	L929 fibroblasts, human nasoseptal chondrocytes (hNC; cell-laden)	Biocompatible and a suitable material for cell culture	Cartilage tissue engineering	[45]
Enzymatic CNF	CNF + alginate; CNF + hyaluronic acid	Hydrogel DIW	Pluripotent stem cells	NFC/A bioinks were suitable for bioprinting iPSCs to support cartilage production in co-culture with irradiated chondrocytes	To repair damaged cartilage in joints	[56]
CM-CNF	CNF + Bacterial cellulose (culture medium)	Hydrogel DIW	Fibroblast cells	Healthy growth	Artificial blood vessels and engineered vascular tissue scaffold	[97]
CM-CNF	Methyltrimethoxysilane hydrophobic CNF matrix-assisted	Hydrogel DIW	A549 lung cancer cells	Sustained healthy cell growth	Open cell culture platform and drug test	[98]
CM-CNF	CNF, CNF/carbon nanotubes	Hydrogel DIW	SH-SHY5Y human neuroblastoma cells	Pure CNF materials are not cytotoxic	Neural tissue engineering	[99]
TEMPO-CNF	CNF + Alginate/Ca^2+^	Hydrogel DIW	L929 mouse fibroblasts	The reduction of cytotoxicity as the ash content of the pulps and CNFs was reduced	Wound dressing devices	[100]
TEMPO-CNF	CNF, TEMPO-CNF, Or acetylated TEMPO-CNF	Hydrogel DIW	Cardiac myoblast cells	Enabled the proliferation and attachment of cells	Cellular processes and tissue engineering	[101]
TEMPO-CNF	CNF + galactoglucomannan methacrylate	Hydrogel DIW	Human dermal fibroblast (HDF) cells and pancreatic tumor cell line SW-1990 cells	Support the principal cell behaviours including cell viability, adhesion, and proliferation	Tissue engineering, cancer cell research, and high-throughput drug screening	[49]
TEMPO-CNF	CNF + gelatin methacrylate	Hydrogel DIW	3T3 fibroblasts cells	Promoted proliferative activity of 3T3 fibroblasts	Wound healing	[48]
TEMPO-CNF	CNF + gelatin methacrylamide	Hydrogel DIW, cell-laden	NIH 3T3 fibroblast cell-laden	No cytotoxicity, high cell viability	Biomedical scaffolds	[57]
CNC	CNC + gelatin	Hydrogel DIW	3T3 fibroblast cells	Support the growth and proliferation of 3T3 cells	Tissue engineering	[102]
CNC	CNC-gelatin conjugates	Hydrogel DIW	Human breast cancer MCF-7 cells	Not cytotoxic	Tissue engineering and regenerative medicine	[103]
CNC	CNC + oxidised dextran/gelatin	Hydrogel DIW	3T3, CCK-8 and Hoechst 33342/PI double-staining assays	Support cell growth and proliferation	Tissue repair	[77]
CNC	CNC + yeast cell + binder (PEGDA) + photo initiator	Viscous paste DIW, cell-laden	Yeast cell-laden	Long-term viability	Microbial biocatalysts, bioremediation	[104]
